# High Prevalence of Colistin Resistance and *mcr-1* Gene in *Escherichia coli* Isolated from Food Animals in China

**DOI:** 10.3389/fmicb.2017.00562

**Published:** 2017-04-04

**Authors:** Xianhui Huang, Linfeng Yu, Xiaojie Chen, Chanping Zhi, Xu Yao, Yiyun Liu, Shengjun Wu, Zewen Guo, Linxian Yi, Zhenling Zeng, Jian-Hua Liu

**Affiliations:** National Reference Laboratory of Veterinary Drug Residues, College of Veterinary Medicine, South China Agricultural UniversityGuangzhou, China

**Keywords:** *Escherichia coli*, colistin, food animals, resistance, *mcr-1*

## Abstract

The objective of this study was to determine the minimal inhibitory concentration of colistin for *Escherichia coli* from food animals and the possible underlying colistin resistance mechanisms. During 2007–2014, 4,438 *E. coli* isolates of food animal origins were collected. The susceptibility of colistin was tested by the agar dilution method. Mutations in *pmrA, pmrB*, and *mgrB* and the presence of *mcr-1* gene were determined by PCR and DNA sequencing. Complementation experiments were carried out to evaluate the contribution of the mutations to colistin resistance. There was a high frequency of colistin resistance in *E. coli* from pigs on farm (24.1%) and at slaughter (24.3%) in 2013–2014, followed by chickens on farm (14.0%) and at slaughter (9.5%). The resistance frequency of *E. coli* in cow isolates was the lowest (0.9%). MIC distribution for colistin showed that most isolates (75.2%) were distributed at 0.25 mg/L–0.5 mg/L, followed by 4 mg/L–8 mg/L (16.8%). Compared with the isolates from pigs and chickens recovered during 2013–2014, *E. coli* isolates collected during 2007–2008 (5.5%) and 2010–2011 (12.4%) showed significantly lower frequency of colistin resistance (*P* < 0.05). DNA sequencing and complementation experiments failed to detect any insertion inactivation or mutation in *pmrA, pmrB*, and *mgrB* associated with colistin resistance. However, 91.0% colistin-resistant isolates were positive for *mcr-1*. The high frequency of colistin resistance and *mcr-1* gene among *E. coli* isolates from food animals in China urged the need to minimize potential risks of colistin resistance development and the spread of *mcr-1* gene.

## Introduction

The rising prevalence of multidrug-resistant (MDR) gram-negative Enterobacteriaceae (GNB), especially carbapenem- resistant, has resulted in a renewed interest in polymyxins, especially polymyxin E (colistin), for the management of gram-negative infections in many countries (Falagas and Michalopoulos, 2006; Cassir et al., 2014). Despite their relatively recent reintroduction in clinical practice, reports on colistin resistant isolates are on the rise (Falagas et al., 2010; Olaitan et al., 2014a). Resistance to polymyxins has been traditionally regarded as occurring via mutations in genes regulating the synthesis of LAra4N (Falagas et al., 2010; Olaitan et al., 2014b). However, we recently described for the first time the emergence of plasmid-mediated colistin resistance gene, *mcr-1*, which now has been identified in several Enterobacteriaceae species from various sources (environment, food, animal and humans) (Liu et al., 2016).

Colistin has been used in veterinary medicine through prophylactic or metaphylactic practices, but the prevalence of colistin resistance in bacteria isolated from animals in many countries was still low (Kempf et al., 2013; Wasyl et al., 2013; Quesada et al., 2014). In China, colistin has been widely used in veterinary medicine, especially in swine and poultry for many years. We previously detected high prevalence of *mcr-1* among *E. coli* isolates from pigs at slaughter in Guangzhou (Liu et al., 2016). Soon after, *mcr-1* gene has been reported to be present in Enterobacteriaceae from animals, food and humans worldwide (Schwarz et al., 2001; Quan et al., 2017; Wang et al., 2017a). However, little is known about the prevalence of colistin resistance and *mcr-1* gene among commensal *E. coli* isolates from other food animals in China. The aim of this study was to investigate the frequency of colistin resistance among commensal *E. coli* isolates from farm animals (chicken, cattle, and pig) and food animals at slaughter recovered from 12 provinces of China and to determine the possible underlying mechanisms among part of colistin-resistant isolates.

## Materials and Methods

### Origin of *E. coli* Isolates

Cloacal samples from chickens (laying hens, chickens, and broilers) and rectal swabs from pigs (piglets, weaned pigs, fattening pigs, and sows) and cattle were collected from 107 food animal farms located in different geographic areas of China (Guangdong, Henan, Jiangxi, Ningxia, Jilin, Qinghai, Sichuan, Shanghai, Jiangsu, Shandong, Beijing, and Neimeng) from May 2013 to August 2014 (**Table 1**). Animals were randomly selected for sampling on each farm based on their age and stage of production. Ten to thirty samples per stage of production per farm were collected. In addition, cecal contents of chickens from seven farmers markets and two live-bird markets and rectal swabs of pigs from two live pig markets and eight abattoirs located in Guangdong, Henan, Shandong, Liaoning, and Sichuan province were collected at slaughter between April 2013 and August 2014. No more than five animal samples per farm were analyzed. All samples were seeded on MacConkey agar plates and were incubated at 37°C for 24 h. One presumptive colony with typical *E. coli* morphology and size was selected and then inoculated on eosin-methylene blue agar. After incubation, suspected *E. coli* colony was identified using classical biochemical methods. In addition, 349 *E. coli* isolates (91 were from chicken during 2007–2008, 86 from chicken during 2010–2011, and 172 from pigs during 2010–2011) from healthy food animals mentioned in our previous study were also included in this study for comparison (Yang et al., 2014).

**Table 1:** Prevalence of colistin resistance among *Escherichia coli* isolates of different origins.

Animals	Farm number	Samples	Number of isolates	Number of colistin-resistant isolates (%)
**2013–2014**				
Laying hens	21	357	295	25 (8.5)
Broilers	43	886	611	102 (16.7)
Chickens	6	90	67	9 (13.4)
All farm chickens	47	1333	973	13 (14.0)
Chickens at slaughter		456	325	31 (9.5)
Piglets	15	275	246	57 (23.2)
Weaned pigs	12	180	150	97 (64.7)
Fattening pigs	32	713	664	141 (21.2)
Sows	24	361	332	26 (7.8)
All farm pigs	46	1529	1392	335 (24.1)
Pigs at slaughter		1200	1063	258 (24.3)
Cows	13	370	336	3 (0.9)
Total		4888	4089	763 (18.7)
**2007–2008**				
Farm chickens			91	5 (5.5)
**2010–2011**				
Farm chickens			86	10 (11.6)
Farm pigs			172	22 (12.8)

### Antimicrobial Susceptibility Testing

The minimal inhibitory concentration (MIC) of colistin was determined by the agar dilution method according to the protocols recommended in M100-S25 of the (Clinical and Laboratory Standards Institute, 2013). For isolates from pigs at slaughter, MICs of ampicillin, cefotaxime, imipenem, gentamicin, amikacin, neomycin, apramycin, florfenicol, tetracycline, ciprofloxacin, and fosfomycin were also determined. The results were interpreted according to epidemiological cut-off (ECOFF) values recommended by EUCAST^1^ (colistin, florfenicol, and neomycin) and the interpretative criteria recommended by CLSI (M100-S25) (ampicillin, cefotaxime, gentamicin, amikacin, fosfomycin, and ciprofloxacin) (Clinical and Laboratory Standards Institute, 2013).

Statistical significance for the comparison of resistance prevalence data was determined by the χ^2^ test. *P* values less than 0.05 were considered statistically significant.

### PCR Amplification and Sequencing

A total of 200 colistin-resistant *E. coli* isolates of different origins (127 from pigs, 70 from chickens, and 3 from cows) were randomly selected for PCR amplification of *mcr-1* (Liu et al., 2016). In addition, 50 of them were randomly selected for sequencing for genes encoding PmrA, PmrB, and MgrB. *pmrA* were amplified using primers described previously (Quesada et al., 2014). The primers used for amplification of entire *mgrB* and *pmrB* genes were as follows: EmgrB-F (5′- CCGCTGAGTAATAATCCTAT -3′) and EmgrB-R (5′- TACAACCAAAGACGCAAT -3′), EpmrB-F (5′- ATAAGCTGAAACGGATGGC -3′) and EpmrB-R (5′- CATAATAATCAGGGCGAAAGT -3′). PCR products of *pmrA, pmrB*, and *mgrB* were sequenced and the nucleotides and deduced protein sequences were analyzed at the National Center for Biotechnology Information web site^2^. In addition, the *pmrA, pmrB*, and *mgrB* sequences of five colistin-susceptible *E. coli* isolates were determined as control.

### Complementation Experiments

The wild-type *mgrB* and *pmrB* genes from an *E. coli* reference strain ATCC 25922 (colistin MIC of 0.25 μg/ml) were amplified by PCR using primers EmgrB-F/EmgrB-R and EpmrB-F/EpmrB-R, respectively. The non-coding mdh sequence was amplified by PCR using primers described previously (Jayol et al., 2014). The PCR products were cloned into the plasmid pCR-BluntII-TOPO (Invitrogen) encoding resistance to kanamycin and zeocin. The resulting plasmids pTOPO-mgrB, pTOPO-pmrB, and pTOPO-mdh were separately transformed into *E. coli* TOP10 strains by electroporation. Transformants were selected by on Mueller–Hinton agar supplemented with 50 mg/L of kanamycin. The recombinant plasmids were isolated and transformed into electrocompetent colistin-resistant *E. coli* with *mgrB* mutation (pTOPO-mgrB) or *E. coli* with *pmrB* mutation (pTOPO-pmrB) via electroporation. The transformants were selected on Mueller–Hinton agar supplemented with zeocin (25 mg/L) and the presence of the cloned gene was confirmed by PCR. The colistin MICs of the transformants were determined by the agar dilution method.

## Results

### Antimicrobial Susceptibility

**FIGURE 1:**
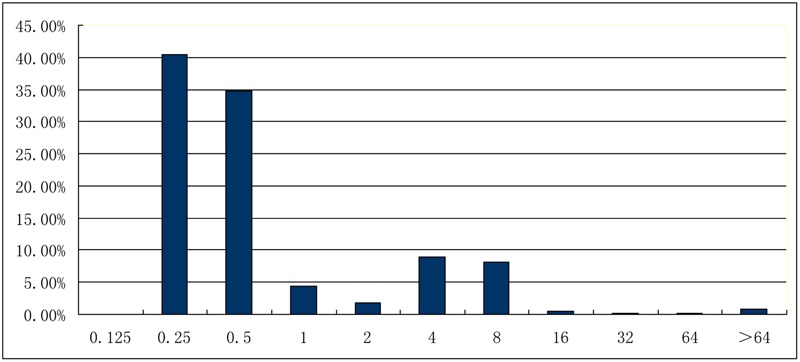
**Distribution of minimal inhibitory concentrations (mg/L) of *Escherichia coli* isolates toward colistin**.

Overall, 4089 commensal *E. coli* collected from 973 chickens on farm, 1392 pigs on farm, 325 chickens at slaughter, 1063 pigs at slaughter, and 336 cows on farm during 2013–2014 were recovered from 4888 samples. Among them, 763 (18.7%) isolates showed resistance to colistin (MIC ≥ 4 mg/L) (**Table 1**). The MIC values were shown in **Figure 1**. MICs of most isolates (75.2%) were distributed at 0.25 mg/L–0.5 mg/L, followed by 4 mg/L–8 mg/L (16.8%). Only MICs of 6.1% isolates and 1.9% isolates were distributed at 1 mg/L–2 mg/L and ≥16 mg/L, respectively.

There was a high frequency of colistin resistance in *E. coli* from pigs on farm (24.1%) and at slaughter (24.3%), followed by chickens on farm (14.0%) and at slaughter (9.5%). The resistance frequency of cow isolates was the lowest (0.9%). Compared with the isolates recovered during 2013-2014, *E. coli* isolates collected during 2007–2008 and 2010–2011 showed significantly lower frequency of colistin resistance (*P* < 0.05, **Table 1**).

Of the 258 colistin resistant isolates from pig at slaughter, 76.7% showed resistance to 3–9 other antimicrobial agents, including tetracycline (93.8%), ampicillin (79.5%), florfenicol (64.3%), cefotaxime (13.2%), neomycin (56.6%), gentamicin (29.1%), ciprofloxacin (28.3%), apramycin (12.4%), fosfomycin (7.8%), and amikacin (0.4%) (**Table 2**). The frequencies of antimicrobial resistance to other antimicrobial agents among colistin resistant isolates were significantly higher than those of colistin susceptible isolates (*P* < 0.01), except to amikacin and gentamicin. All of the isolates were susceptible to imipenem.

**Table 2:** Comparison of antimicrobial susceptibility of colistin-susceptible isolates and colistin-resistant isolates from pigs at slaughter.

Antimicrobial agents	Colistin-susceptible isolates (*n* = 805) (%)	Colistin-resistant isolates (*n* = 258) (%)	chi-square value	*P*-value
Ampicillin	65.30	79.50	18.126	< 0.0001
Cefotaxime	6.60	13.20	11.307	0.0008
Amikacin	0.90	0.90	0	1.000
Gentamicin	23.90	29.10	2.8293	0.093
Apramycin	4.5	12.40	20.5248	< 0.0001
Neomycin	32.60	56.60	47.52	< 0.0001
Tetracycline	84.80	93.80	293.998	< 0.0001
Florfenicol	47.50	64.30	22.31	< 0.0001
Fosfomycin	2.10	7.80	18.5003	< 0.0001
Ciprofloxacin	17.60	28.30	13.7470	0.0002

### *mcr-1* Detection and Sequence of *pmrA, pmrB*, and *mgrB* Genes

Of the 200 randomly seiected colistin-resistant isolates, 182 (91.0%) were positive for *mcr-1*. The sequences of the *pmrA, pmrB*, and *mgrB* genes known to be involved in polymyxin resistance were determined in 50 isolates. For PmrA, no amino acid substitution was observed among the 50 isolates except one isolate that had the G144S substitution. However, G144S substitution was found to be present in colistin-susceptible isolates (Quesada et al., 2014). For MgrB, 4 isolates from different regions possessed D31G substitution. For PmrB, one isolate had two amino acid substitutions (T246I and D282N). However, the MICs of colistin remained unchanged upon transformation with plasmid pTOPO-mgrB or pTOPO-pmrB.

## Discussion

Despite the frequent use of colistin in animal farming for over 50 years, the occurrence of colistin resistance among *E. coli* strains isolated from food animals remains low (<1%) (Kempf et al., 2013; Kieffer et al., 2015). However, in this study, we found a very high prevalence of colistin resistance (18.7%) among commensal *E. coli* isolates from food animals, especially pigs. The frequency of resistance in commensal intestinal *E. coli* is considered to be a good marker for the selection pressure exerted by antibiotic use in the host animals and the resistance problems to be predicted in pathogenic bacteria (van den Bogaard and Stobberingh, 2000). This high prevalence of colistin resistance may be due to the increasing use of colistin in food animals in recent years. Our previous studies showed that most *E. coli* strains from chicken and pigs in China showed resistance to fluoroquinolones and florfenicol, and over 20% isolates exhibited resistance to third-generation cephalosporins, amikacin and fosfomycin (Chen et al., 2014; Rao et al., 2014). Thus, in recent years, the lack of effective drugs against *E. coli* might be attributed by the increased consumption of colistin in veterinary medicine, especially in piglets which are frequently treated with colistin sulphate for colibacillosis. This high selective pressure might result in the highest prevalence (64.7%) of colistin resistance among *E. coli* isolates from weaned piglets found in this study. Compared with pig and chicken isolates, the prevalence of colistin resistance among *E. coli* from cows was very low (0.9%) which might be associated with the infrequent use of this drug on dairy-farm.

To determine whether there was an increase of colistin resistance from 2007 to 2014, *E. coli* isolates collected in our previous study were reviewed for colistin resistance. By comparison, colistin resistance among *E. coli* isolated from chicken raised nearly three times from 2007/2008 to 2013/2014 and that among *E. coli* isolated from pigs raised nearly two times from 2010/2011 to 2013/2014. Though we could not obtain the amount of colistin consumption on each farm sampled in this study, data from China Veterinary Drug Association showed that the volume of colistin sales increased significantly from 2011 to 2013 (China Veterinary Drug Association, 2014). Taken together, our results revealed that colistin resistance in food animals was correlated with the consumption of colistin.

Interestingly, MICs of most colistin- resistant isolates were 4 or 8 mg/L. The emergence and spread of *E. coli* with low level of colstin resistance (MIC = 4 or 8) might lead to the treatment failure of diarrhea with standard colistin dosage (2–20 mg/kg, ppm). Thus, farmers have to illegally use medicated feed added with increased dosage of colistin (80–100 ppm) to prevent diarrhea in piglets (personal communication).

Colistin resistance among commensal *E. coli* isolates recovered from pigs at slaughter was also worrisomely high. These resistant bacteria might contaminate meat during slaughtering procedures and transfer to humans by food chain or to workers via direct animal contact as indicated in some previous studies (Angulo et al., 2004; Liebana et al., 2013). Recently, several studies suggested the possibility of the transference MCR-1-producing Enterobacteriaceae to humans via food chain (Campos et al., 2016; Carnevali et al., 2016; Figueiredo et al., 2016; Wang et al., 2017b). Hence it is urgent to limit the usage of polymyxins (colistin) in veterinary medicine especially as feed additives in China. Fortunately, following our discovery of *mcr-1*, the Chinese Government has banned the use of colistin in animal feed since Nov 1, 2016 (Walsh and Wu, 2016).

Similar to our previous results, colistin resistance is mainly caused by *mcr-1* gene. Quesada et al. have recently found mutations in PmrAB that confer resistance to polymyxins in *E. coli* (Quesada et al., 2014). However, in this study, we failed to detect any meaningful mutation in *pmrAB* and *mgrB* conferring resistance to colistin. Further studies are needed to understand the possible mechanism mediating colistin resistance among *mcr-1*-negative isolates.

## Conclusion

We have detected a high prevalence of colistin resistance and *mcr-1* gene in *E. coli* from food animals. Though colistin exhibited high antimicrobial activities against GNB, including *E. coli, A. baumannii, Pseudomonas aeruginosa* isolates, and *K. pneumonia* in human (Chen et al., 2015), the frequent presence of *mcr-1*-positive *E. coli* and in food animals might be a threat to human. As colistin is the last therapeutic option against infections caused by MDR GNB, careful monitoring of the evolution of colistin resistance and the spread of *mcr-1* gene in isolates from humans in China is urgently needed.

## Author Contributions

Conceived and designed the experiments: J-HL, XH, and ZZ. Performed the experiments: XH, LY, XC, CZ, XY, YL, SW, ZG, and LY. Analyzed the data: J-HL, XH, LY, YL, and ZZ. Wrote the paper: J-HL and XH.

## Conflict of Interest Statement

The authors declare that the research was conducted in the absence of any commercial or financial relationships that could be construed as a potential conflict of interest.

## Footnotes

1 http://www.eucast.org

2 http://www.ncbi.nlm.nih.gov
